# Burosumab vs conventional therapy in children with X-linked hypophosphatemia: results of the open-label, phase 3 extension period

**DOI:** 10.1093/jbmrpl/ziad001

**Published:** 2024-01-04

**Authors:** Leanne M Ward, Wolfgang Högler, Francis H Glorieux, Anthony A Portale, Michael P Whyte, Craig F Munns, Ola Nilsson, Jill H Simmons, Raja Padidela, Noriyuki Namba, Hae Il Cheong, Etienne Sochett, Koji Muroya, Hiroyuki Tanaka, Pisit Pitukcheewanont, Gary S Gottesman, Andrew Biggin, Farzana Perwad, Angel Chen, John Lawrence Merritt II, Erik A Imel

**Affiliations:** Department of Pediatrics, Faculty of Medicine, University of Ottawa, Ottawa, Ontario, K1H 8L1, Canada; Department of Paediatrics and Adolescent Medicine, Johannes Kepler University Linz, 4040 Linz, Austria; Institute of Metabolism and Systems Research, University of Birmingham, Edgbaston, Birmingham, B15 2TT, United Kingdom; Department of Surgery, Pediatrics, and Human Genetics, Shriners Hospitals for Children and McGill University, Montreal, Quebec, H4A 0A9, Canada; Department of Pediatrics, University of California, San Francisco, CA, 94158, United States; Shriners Hospitals for Children St Louis, St Louis, MO, 63110, United States; Faculty of Medicine, The University of Queensland, Brisbane, Queensland, 4072, Australia; Faculty of Medicine, Child Health Research Centre, The University of Queensland, Brisbane, Queensland, 4072, Australia; Department of Endocrinology and Diabetes, Queensland Children’s Hospital, Brisbane, Queensland, 4072, Australia; Division of Pediatric Endocrinology, Department of Women’s and Children’s Health, Center for Molecular Medicine, Karolinska Institutet and University Hospital, SE-17176 Stockholm, Sweden; Department of Pediatrics, School of Medical Sciences, Örebro University and University Hospital, S-701 82 Örebro, Sweden; Division of Endocrinology and Diabetes, Department of Pediatrics, Vanderbilt University School of Medicine, Vanderbilt University, Nashville, TN, 37232, United States; Department of Paediatric Endocrinology, Royal Manchester Children’s Hospital, Manchester, M13 9WL, United Kingdom; Department of Pediatrics, Osaka Hospital, Japan Community Healthcare Organization, Osaka, 553-0003, Japan; Osaka University Graduate School of Medicine, Osaka, 565-0871, Japan; Department of Pediatrics, Hallym University Sacred Heart Hospital, Dongan-gu, Anyang, 14068, South Korea; Department of Pediatrics, Hospital for Sick Children, Toronto, Ontario, M5G 1E8, Canada; Department of Endocrinology, Kanagawa Children’s Medical Center, Yokohama, Kanagawa 232-0066, Japan; Division of Pediatrics, Okayama Saiseikai General Hospital Outpatient Center, Kita-ku, Okayama, 700-8511, Japan; Center of Endocrinology, Diabetes and Metabolism, Children’s Hospital of Los Angeles, Los Angeles, CA, 90027, United States; Shriners Hospitals for Children St Louis, St Louis, MO, 63110, United States; Department of Endocrinology, The University of Sydney Children’s Hospital Westmead Clinical School, The Children’s Hospital at Westmead, Westmead, New South Wales, 2145, Australia; Department of Pediatrics, University of California, San Francisco, CA, 94158, United States; Ultragenyx Pharmaceutical Inc., Novato, CA, 94949, United States; Ultragenyx Pharmaceutical Inc., Novato, CA, 94949, United States; Department of Medicine, Indiana University School of Medicine, Indianapolis, IN, 46202, United States

**Keywords:** burosumab, FGF23, phosphate, rare disease, x-linked hypophosphatemia

## Abstract

In a randomized, open-label phase 3 study of 61 children aged 1–12 years old with X-linked hypophosphatemia (XLH) previously treated with conventional therapy, changing to burosumab every 2 weeks (Q2W) for 64 weeks improved the phosphate metabolism, radiographic rickets, and growth compared with conventional therapy. In this open-label extension period (weeks 64–88), 21 children continued burosumab Q2W at the previous dose or crossed over from conventional therapy to burosumab starting at 0.8 mg/kg Q2W with continued clinical radiographic assessments through week 88. Efficacy endpoints and safety observations were summarized descriptively for both groups (burosumab continuation, *n* = 6; crossover, *n* = 15). At week 88 compared with baseline, improvements in the following outcomes were observed in the burosumab continuation and crossover groups, respectively: mean (SD) RGI-C rickets total score (primary outcome), +2.11 (0.27) and +1.89 (0.35); mean (SD) RGI-C lower limb deformity score, +1.61 (0.91) and +0.73 (0.82); and mean (SD) height *Z*-score + 0.41 (0.50) and +0.08 (0.34). Phosphate metabolism normalized rapidly in the crossover group and persisted in the continuation group. Mean (SD) serum alkaline phosphatase decreased from 169% (43%) of the upper limit of normal (ULN) at baseline to 126% (51%) at week 88 in the continuation group and from 157% (33%) of the ULN at baseline to 111% (23%) at week 88 in the crossover group. During the extension period, treatment-emergent adverse events (AEs) were reported in all 6 children in the burosumab continuation group and 14/15 children in the crossover group. The AE profiles in the randomized and extension periods were similar, with no new safety signals identified. Improvements from baseline in radiographic rickets continued in the extension period among children with XLH who remained on burosumab. Children who crossed over from conventional therapy to burosumab demonstrated a rapid improvement in phosphate metabolism and improved rickets healing over the ensuing 22 weeks.

## Teaser

X-linked hypophosphatemia (XLH) is a heritable disorder with key clinical features caused by low phosphate levels in children, including rickets, poor growth, and leg bowing. Traditionally, XLH has been treated with multiple daily doses of phosphate and an active form of vitamin D. “Burosumab” is a novel antibody therapy that blocks a phosphate-wasting hormone called FGF23. Over 64 weeks,this therapy has shown to be better able to restore normal phosphate levels, heal rickets, and improve growth and leg bowing compared with traditional therapy in children 1–12 years of age. In the current study, children originally treated with burosumab or conventional therapy for 64 weeks with underwent a further study up to 88 weeks. Here, improvements from baseline in rickets continued between 64 and 88 weeks among children who remained on burosumab. Children who crossed over from conventional therapy to burosumab demonstrated a rapid improvement in phosphate metabolism and improved rickets healing, affirming the findings of the pivotal 64-week trial. As in the initial 64-week trial, burosumab was well tolerated in children who continued burosumab through 88 weeks, and in those who crossed over from conventional therapy. These data provide additional support for the use of burosumab to treat pediatric XLH.

## Graphical Abstract

**Graphical Abstract:**
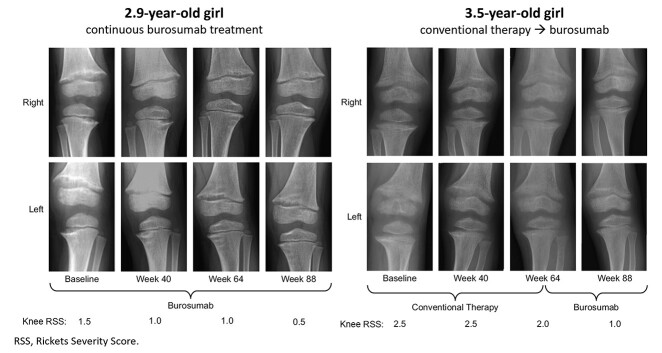

Following the 64-week, open-label, phase 3 study of burosumab vs conventional therapy in children aged 1–12 years with X-linked hypophosphatemia (NCT02915705), participants could enter the 22-week extension period in which all participants received burosumab treatment. Both those who continued to receive burosumab (top panel) and those who started burosumab during the extension period (bottom panel) showed improvements in Knee Rickets Severity Scores from burosumab initiation. These improvements were supported by similar changes in Radiographic Global Impression of Change Rickets Total score, height *Z*-score, and serum phosphorus and alkaline phosphatase levels.

## Introduction

X-linked hypophosphatemia (XLH) is a rare musculoskeletal disorder caused by loss-of-function mutations in the Phosphate Regulating Endopeptidase Homolog X-linked (*PHEX*) gene, resulting in excessive plasma levels of fibroblast growth factor 23 (FGF23).^1^^,^^2^ Increased circulating FGF23 inhibits renal phosphate reabsorption and synthesis of 1,25-dihydroxyvitamin D (1,25[OH]_2_D). These disruptions cause hypophosphatemia and defective bone mineralization that result in rickets, osteomalacia, skeletal deformities, short stature, pain, gait abnormalities, and impaired physical function.^3^

Although conventional therapy with phosphate salts and active vitamin D may alter the clinical course of XLH in children and adults, symptoms can persist. Long-term use may be associated with complications, such as nephrocalcinosis and hyperparathyroidism.^4-6^ Burosumab is a fully human IgG1 monoclonal antibody to FGF23 approved for the treatment of XLH.^7-9^ In 2019, we reported the results from a randomized, open-label phase 3 study in children 1–12 years old with XLH receiving conventional therapy who showed significantly greater improvement in phosphate metabolism, rickets healing, linear growth, lower limb deformity, and mobility when switched to burosumab compared with maintaining conventional therapy.^10^

The purpose of this report is to describe the efficacy and safety of burosumab through the open-label extension period of the original phase 3 study (weeks 64–88) in which children with XLH continued to receive burosumab or crossed over from conventional therapy to burosumab. The objective was to assess the longer term safety and impact of FGF23 blockade with burosumab beyond 64 weeks on key clinical outcomes, including phosphate metabolism, rickets healing, lower limb deformity, and growth.

## Materials and methods

### Participants

Full details of the randomized, open-label phase 3 trial (ClinicalTrials.gov, NCT02915705) preceding this extension period are published^10^ and provided in the study protocol. Inclusion criteria included ages 1–12 years, fasting serum phosphorus <3.0 mg/dL (0.97 mmol/L), a *PHEX* mutation or variant of unknown significance in the child or a family member consistent with an X-linked dominant inheritance pattern, a total rickets severity score (RSS) ≥2.0,^11^ and prior treatment with conventional therapy for ≥6 consecutive months if aged <3 years or ≥ 12 months if aged ≥3 years.

Exclusion criteria included Tanner stage 4 or greater, height above 50th percentile for age and sex based on country-specific norms, growth hormone therapy in the 12 months before screening, plasma parathyroid hormone >180 pg/mL (>19 pmol/L), hypocalcemia or hypercalcemia, renal ultrasound indicating nephrocalcinosis grade 4 (on a scale of 0–4), and planned orthopedic surgery. Parents or guardians provided written informed consent for their children to participate, with the child’s written assent according to the local guidelines. The institutional review board at each participating site approved the protocol. An independent committee monitored safety during the study that was conducted in accordance with the Declaration of Helsinki and the Good Clinical Practice guidelines developed at the International Conference on Harmonization of Technical Requirements for Registration of Pharmaceuticals for Human Use.

### Study design and treatment

At the beginning of the 64-week randomized period of the trial,^10^ following a 7-day conventional therapy washout period, children were randomly assigned 1:1 by the Interactive Web Response System (version 1.3.6.26) in blocks of 4 to receive subcutaneous burosumab starting at 0.8 mg/kg every 2 weeks (Q2W) or to resume conventional therapy titrated by the investigator per published recommendations (oral phosphate 20–60 mg/kg per day and alfacalcidol 40–60 ng/kg per day or calcitriol 20–30 ng/kg per day).^5^^,^^6^ The dose of burosumab was increased to 1.2 mg/kg Q2W if two consecutive pre-dose, fasting serum phosphorus concentrations were < 3.2 mg/dL (<1.03 mmol/L), and if serum phosphorus had not increased from baseline by at least 0.5 mg/dL (<0.16 mmol/L) in a single measurement. Randomization was stratified by rickets severity (RSS ≤ 2.5 vs >2.5),^11^ age (<5 vs ≥5 years), and geographic region (Japan vs rest of world).

After completion of the randomized period, children in Europe, the United States, Canada, and Australia were eligible to enter the extension period (planned duration, weeks 64–124) to continue burosumab at the previously received dose (ie, burosumab continuation group) or crossover to open-label burosumab following a 7-day washout (ie, crossover group). In the crossover group, burosumab was initiated at week 66 at 0.8 mg/kg Q2W and could be increased to 1.2 mg/kg Q2W, as described above. The extension period ended when burosumab became commercially available in Europe, the United States, and Australia in September 2018 and in Canada in June 2019. Consequently, data are reported for children with radiographic assessments through week 88 of the extension period.

### Outcomes

The primary endpoint of the randomized period was the change from baseline to week 40 in the radiographic appearance of rickets using the Radiographic Global Impression of Change (RGI-C) total score (referred to hereafter as the “RGI-C rickets total score”) assessed by three radiologists blinded to treatment group, whose scores were averaged; the secondary endpoints included the change in radiographic appearance from baseline to week 64 assessed by RGI-C rickets total score.^10^ The RGI-C rickets total score included an independent, qualitative assessment of the change in skeletal abnormalities (eg, physeal or metaphyseal lucency, separation, fraying, and concavity) relative to baseline using wrist and knee radiographs; the RGI-C rickets total score is validated for the assessment of rickets healing in XLH.^10^^,^^12-14^ During the extension period, the RGI-C rickets total score was assessed by comparing baseline radiographs to those at week 88. The change in lower limb deformity (genu varum and valgum), a component of RGI-C, was determined by comparing differences in standing lower limb radiograph pairs at weeks 40, 64, and 88.

The RSS is a validated method that assigns a total score ranging from 0 (no rickets) to 10 (severe rickets) based on the sum of scores from the most severely affected wrist (0–4) and knee (0–6).^10^^,^^11^^,^^15^ The total RSS was assessed at baseline and at weeks 40, 64, and 88 as previously described.

Change from baseline to weeks 24, 40, 64, 76, and 88 in standing height or recumbent length *Z*-scores was calculated using age- and sex-matched normative data from the United States Centers for Disease Control and Prevention and using Tanner’s standard.^10^^,^^16^^,^^17^ Change from baseline to the same time points in growth velocity *Z*-scores was calculated using age- and sex-matched data for children with available pre-study growth records (for the determination of baseline values) using Tanner’s standard.^10^^,^^17^

The 6-minute walk test (6MWT) was used to assess mobility in children aged ≥5 years at baseline and at weeks 24, 40, 64, and 88.^10^^,^^18^ Results were expressed as the percentage predicted for height, age, and sex compared with baseline.

Fasting serum phosphorus, 1,25[OH]_2_D, 25-hydroxyvitamin D, alkaline phosphatase (ALP), and tubular maximum for phosphate reabsorption per glomerular filtration rate (TmP/GFR) and urinary calcium were assessed at baseline, at various timepoints during the randomized period, and at weeks 68, 76, and 88 during the extension period. TmP/GFR was calculated using data from fasting 2-h urine; however, spot urine was used for seven predominantly younger children.

All adverse events (AEs) were tabulated. Nephrocalcinosis was evaluated by renal ultrasonography on an ordinal scale 0 (absent) to 4 (stone formation) by readers blinded to treatment.^19^ Physical examinations were performed regularly throughout the study by the investigators.

### Statistical analysis

The present analysis includes the subset of children originally enrolled in the phase 3 trial who had radiographic assessments available through 88 weeks. The study was not powered to assess within- or between-group changes in clinical outcomes; therefore, results were expressed descriptively as means and SDs.

## Results

### Patients

**Figure 1:**
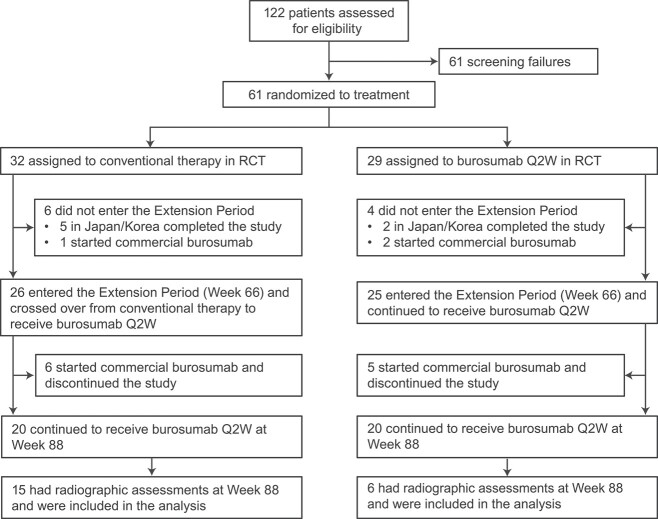
Disposition through week 88 of the extension period.

From September 08, 2016 to July 30, 2018, 61 children received treatment in the randomized period (burosumab, *n* = 29; conventional therapy, *n* = 32) for 64 weeks.^10^ Fifty-one of the 61 children entered the extension period, 11 of whom discontinued due to the availability of commercial burosumab (Figure 1). Twenty-one of the 40 remaining children had available radiographic assessments through week 88 (burosumab continuation, *n* = 6; crossover, *n* = 15) and were included in the present analysis. The demographic and baseline characteristics of the children included in this analysis to 88 weeks are summarized in Table 1. The extension period included six boys (burosumab continuation, *n* = 2; crossover, *n* = 4) and 15 girls (burosumab continuation, *n* = 4; crossover, *n* = 11).

**Table 1:** Demographics and disease characteristics at baseline of the randomized period and at the beginning of the open-label extension period.^a^^,^^b^

	**Beginning of randomized period (baseline)** ^a^		**Beginning of open-label extension period (week 66)** ^a^	
	**Conventional therapy (*n* = 15)**	**Burosumab (*n* = 6)**	**Crossover (*n* = 15)**	**Burosumab continuation (*n* = 6)**
Mean (SD) age, years	6.6 (3.0)	6.3 (3.6)	8.0 (3.0)	7.8 (3.6)
Sex, *n* (%)				
Boys	4 (27)	2 (33)	4 (27)	2 (33)
Girls	11 (73)	4 (67)	11 (73)	4 (67)
Race, *n* (%)				
White	14 (93)	6 (100)	14 (93)	6 (100)
Asian	1 (7)	0	1 (7)	0
Height *Z*-score				
Mean (SD)	−2.07 (1.01)	−2.79 (1.33)	−2.01 (1.02)	−2.43 (1.28)
Median (IQR)	−2.14 (−2.29, −1.44)	−2.64 (−3.20, −2.29)	−2.04 (−2.38, −1.25)	−2.51 (−2.67, −1.90)
Mean (SD) serum phosphorus, mg/dL	2.37 (0.25)	2.28 (0.19)	2.64 (0.43)	3.53 (0.66)
Mean (SD) TmP/GFR, mg/dL	2.07 (0.30)	1.95 (0.34)	1.97 (0.55)	3.57 (0.75)
Mean (SD) serum 1,25(OH)_2_D, pg/mL	36.64 (11.04)	38.00 (21.36)	40.04 (12.07)	61.72 (12.72)
Mean (SD) serum 25(OH)D, ng/mL	36.27 (8.89)	33.47 (5.84)	34.50 (10.26)	36.63 (22.23)
Mean (SD) serum alkaline phosphatase, U/L	497.20 (92.51)	543.50 (103.72)	473.07 (87.37)	410.83 (112.92)
Duration of conventional therapy, years				
Mean (SD)	4.2 (2.8)	4.4 (4.6)	5.5 (2.8)	4.4 (4.6)
Median (IQR)	3.4 (1.7, 6.7)	1.9 (1.6, 7.8)	4.6 (2.9, 8.0)	1.9 (1.6, 7.8)
Mean (SD) total RSS	3.10 (1.06)	3.08 (0.49)	2.30 (1.07)	0.92 (0.49)
XLH-related medical history, *n* (%)				
Bowing of the tibia/fibula	11 (73)	6 (100)	11 (73)	6 (100)
Bowing of the femur	13 (87)	6 (100)	13 (87)	6 (100)
Intoeing	12 (80)	5 (83)	12 (80)	5 (83)
Dental abscesses	5 (33)	3 (50)	5 (33)	3 (50)
Genu valgum	4 (27)	2 (33)	4 (27)	2 (33)
Joint stiffness (limited range of motion)	2 (13)	2 (33)	2 (13)	2 (33)
Craniosynostosis	4 (27)	0	4 (27)	0
Multiple cavities	3 (20)	2 (33)	3 (20)	2 (33)
Chiari malformation	0	0	0	0

1,25(OH)_2_D, 1,25-dihydroxyvitamin D; 25(OH)D, 25-hydroxyvitamin D; RSS, rickets severity score; XLH, X-linked hypophosphatemia.

^a^Data are shown for at the beginning of randomized period (ie, baseline) or at the beginning of the extension period (ie, week 66) for children with available radiographic assessments through week 88, with exception of XLH-related medical history data, which are all from the beginning of the randomized period.

^b^Data are shown for children with available radiographic assessments through week 88.

### Exposure

During the extension period, five of six children in the burosumab continuation group received all planned total doses of burosumab at 0.8 mg/kg Q2W; one child in this group had a protocol-specified dose increase to 1.2 mg/kg Q2W. Thirteen children in the crossover group received all planned total doses of burosumab at 0.8 mg/kg Q2W; two children in this group had protocol-specified dose increases to 1.2 mg/kg Q2W. One child in the crossover group received three doses of burosumab at 1.2 mg/kg Q2W in error and was returned to the initial dose of 0.8 mg/kg Q2W. The highest individual weight-based burosumab dose was 1.27 mg/kg in the randomized period and 1.27 mg/kg in the extension period.

### Efficacy

**Figure 2:**
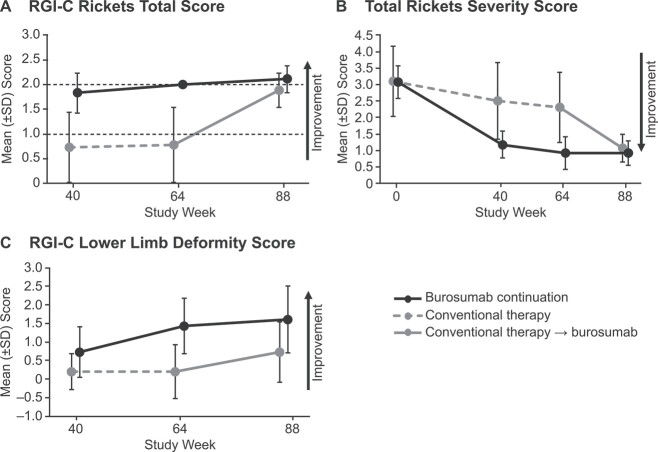
Rickets and bone health evaluations in children with available radiographic assessments through week 88. (A) RGI-C rickets total score at weeks 40, 64, and 88. (B) Total RSS at baseline and weeks 40, 64, and 88. (C) RGI-C lower limb deformity score at weeks 40, 64, and 88. RSS, Rickets Severity Score.

In the burosumab continuation group (*n* = 6), the mean (SD) RGI-C rickets total score at week 64 was +2.00 (0.00), and it was sustained at extension period week 88 with a score of +2.11 (0.27) (Figure 2A). In the crossover group (*n* = 15), the mean (SD) RGI-C rickets total score increased from +0.78 (0.75) at week 64 while on conventional therapy to +1.89 (0.35) at week 88 after switching to burosumab. Improvements in rickets, as assessed by mean (SD) RGI-C wrist and knee scores, also persisted from weeks 40 and 64 to week 88 in the burosumab continuation group and increased from weeks 40 and 64 to week 88 in the crossover group after switching from conventional therapy to burosumab (Supplementary Table 1). The effect of pubertal status on rickets healing among the 21 patients included in this analysis is unclear, as only four had postbaseline Tanner stage >1, of whom three were Tanner stage 1 at baseline.

At week 88, all 6 (100%) children in the burosumab continuation group and 13 of 15 (87%) in the crossover group achieved substantial healing of rickets (defined as an RGI-C rickets total score ≥ +2) compared with 1 of 15 (7%) at week 64 before switching to burosumab. All six (100%) children in the burosumab-continuation group had improvement from baseline to week 88 in all metaphyseal abnormalities at all measured sites except for metaphyseal concavity at the fibula, which was unchanged in one of the three children who had this feature at baseline (Supplementary Table 2). Nearly all 15 children in the crossover group had improvements from baseline to week 88 vs baseline in each of the four distal femur metaphyseal abnormalities (lucency, 100%; separation, 100%; fraying, 93%; concavity, 100%).

**Figure 3:**
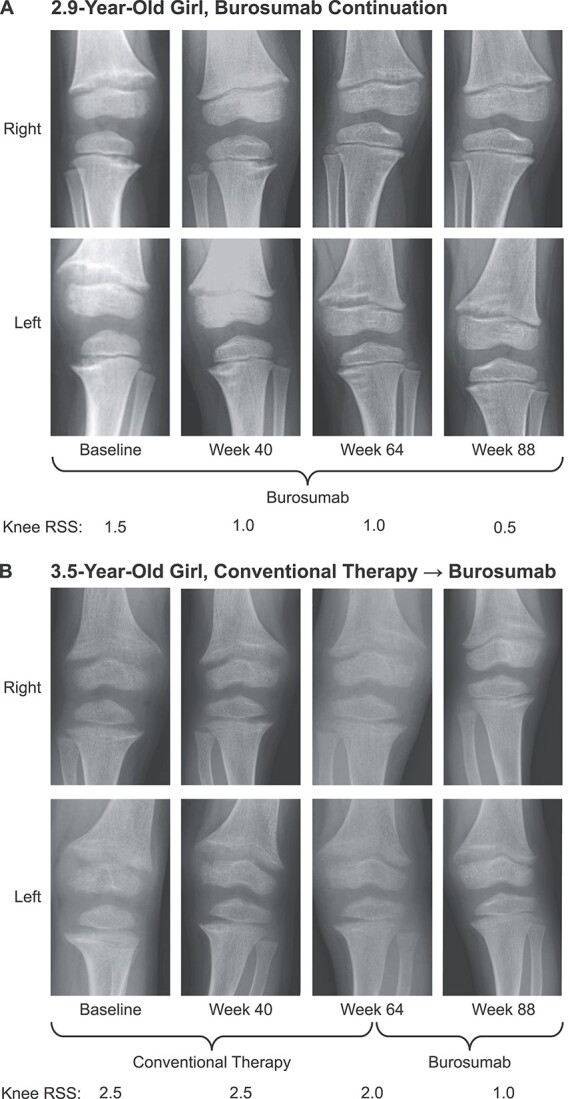
Knee radiographs showing rickets improvement with burosumab treatment. Shown are knee RSS scores and radiographs at baseline and at weeks 40, 64, and 88 in a 2.9-year-old girl in the burosumab continuation group (A) and in a 3.5-year-old girl in the conventional therapy to burosumab crossover group (B). RSS, Rickets Severity Score.

In the burosumab continuation group, 100% of the improvement in mean total RSS over the 88 weeks of the study occurred between baseline and week 64; the improvement in total RSS between baseline (mean [SD]: 3.08 [0.49]) and week 64 (0.92 [0.49]; mean [SD] change from baseline, −2.17 [0.61]) was sustained at week 88 (0.92 [0.38]; mean [SD] change from baseline, −2.17 [0.41]) (Figure 2B). In the crossover group, mean (SD) total RSS improved from 3.10 (1.06) at baseline to 2.30 (1.07) at week 64 (mean [SD] change from baseline, −0.80 [0.94]) while on conventional therapy and further improved to 1.07 (0.42) at week 88 (mean [SD] change from baseline, −2.03 [1.0]) after switching to burosumab. In the crossover group, of the total improvement in mean total RSS that occurred over the 88 weeks of the study, 39% occurred during the 64 weeks of conventional therapy, and 61% occurred during the 22 weeks after switching to burosumab. Radiographic examples of rickets healing with burosumab are provided in Figure 3.

**Figure 4:**
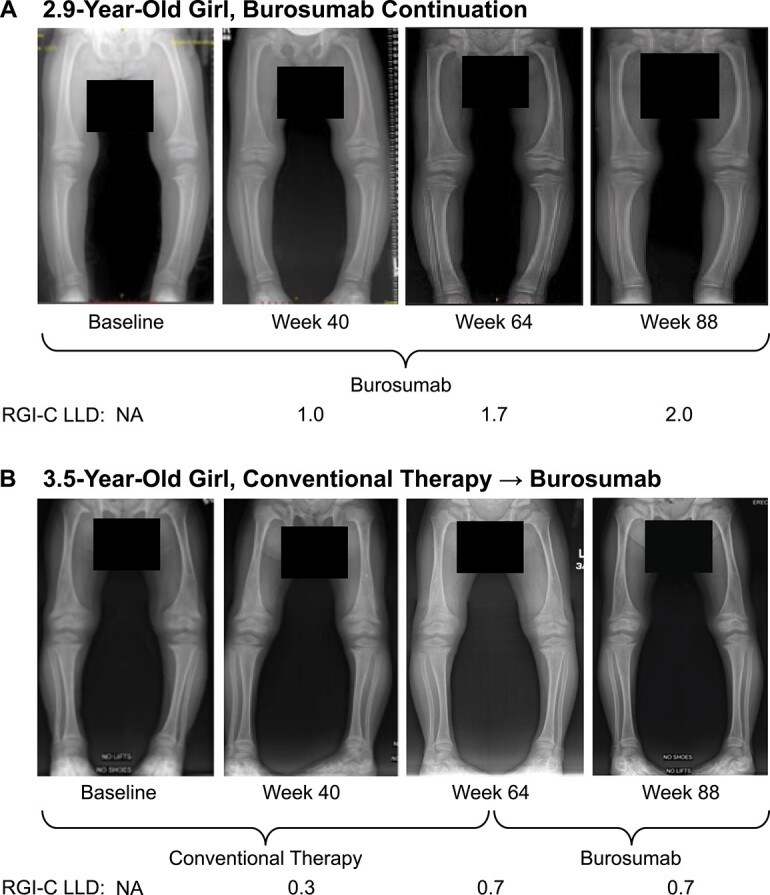
Radiographs showing improvement in lower limb deformity with burosumab treatment. Shown is the lower limb deformity RGI-C at baseline and at weeks 40, 64, and 88 in a 2.9-year-old girl in the burosumab continuation group (A) and in a 3.5-year-old girl in the conventional therapy to burosumab crossover group (B). LLD, lower limb deformity; NA, not applicable; RGI-C, Radiographic Global Impression of Change.

In the burosumab continuation group, improvements in the RGI-C lower limb deformity score at week 64 (mean [SD]: +1.44 [0.75]) were sustained at week 88 (+1.61 [0.91]) (Figure 2C). In the crossover group, the RGI-C lower limb deformity score at week 64 (mean [SD]: +0.20 [0.73]) increased at week 88 (+0.73 [0.82]). Radiographic examples of lower limb deformity healing with burosumab are provided in Figure 4.

**Figure 5:**
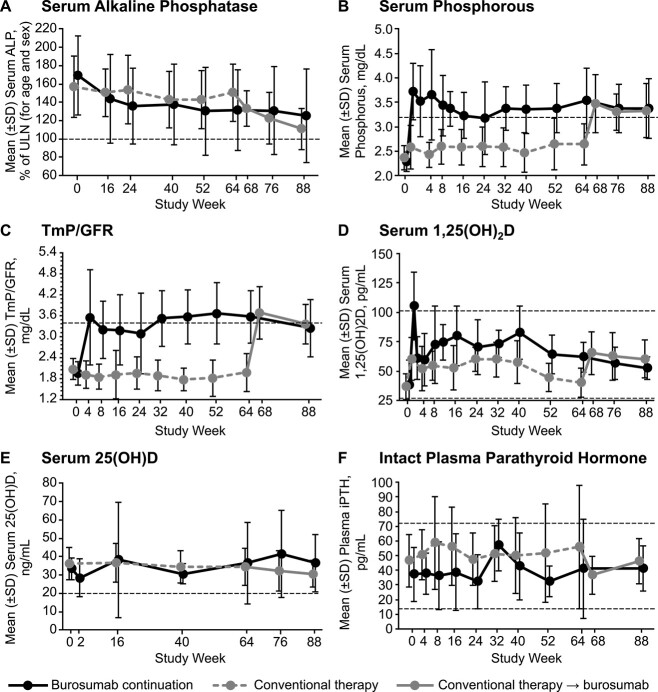
Mean (SD) serum alkaline phosphatase (A), serum phosphorus (B), TmP/GFR (C), serum 1,25(OH)_2_D (D), serum 25(OH)D (E), and plasma intact parathyroid hormone (F) in children with available radiographic assessments through week 88. Dashed lines indicate the upper limit of normal for alkaline phosphatase, the lower limit of normal for serum phosphorus (3.2–6.1 mg/dL), the lower limit of normal for TmP/GFR (3.4–5.8 mg/dL), the lower and upper limits of normal for serum 1,25(OH)_2_D (25.8–101.5 pg/mL), the 25(OH)D status benchmarked to 20 ng/mL, and the lower and upper limits of normal for serum intact parathyroid hormone (14.0–72.0 pg/mL). Alkaline phosphatase is shown as the percent of ULN for age and sex, and the ULN is labeled as 100%, calculated from the following normal ranges: girls 1–4 years, 317 U/L; girls 4–7 years, 297 U/L; girls 7–10 years, 325 U/L; girls 10–15 years, 300 U/L; boys 1–4 years, 383 U/L; boys 4–7 years, 345 U/L; boys 7–10 years, 309 U/L; and boys 10–15 years, 385 U/L. For iPTH, the dashed line indicates the upper limit of the normal range (14–72 pg/mL). 1,25(OH)_2_D, 1,25-dihydroxyvitamin D; 25(OH)D, 25-hydroxyvitamin D; AP, alkaline phosphatase; iPTH, intact parathyroid hormone; TmP/GFR, tubular maximum for phosphate reabsorption per glomerular filtration rate.

Among children in the burosumab continuation group, mean (SD) serum ALP concentrations decreased from 169% (43%) of upper limit of normal (ULN) at baseline to 131% (44%) of ULN at week 64 (mean [SD] change from baseline, −42% [20%]) and to 126% (51%) of ULN at week 88 (−48% [15%]; Figure 5A). In the crossover group, mean (SD) serum ALP decreased from 157% (33%) of ULN at baseline to 151% (31%) of ULN at week 64 (mean [SD] change from baseline, −9.0% [37%]) and to 111% (23%) of ULN at week 88 (−49% [26%]). Although most of the decline in serum ALP occurred by week 64 in the burosumab continuation group, most of the improvement in the crossover group occurred after transition to burosumab. At week 88, 3 of 6 (50%) children in the burosumab continuation group and 5 of 15 (33%) children in the crossover group had serum ALP levels in the normal range for age and sex.

In the burosumab continuation group, mean (SD) fasting serum phosphorous was 2.3 (0.2) mg/dL at baseline, 3.5 (0.7) mg/dL at week 64 (mean [SD] percentage change from baseline, +55.4% [29.8%]), and 3.4 (0.6) mg/dL at week 88 (+48.1% [28.50%]); Figure 5B. In the crossover group, mean (SD) fasting serum phosphorus was 2.4 (0.3) mg/dL at baseline, 2.6 (0.4) mg/dL at week 64 (mean [SD] percentage change from baseline, +12.1% [18.0%]), and 3.3 (0.5) mg/dL at week 88 (+41.0% [18.9%]). Hyperphosphatemia did not occur during the randomized or extension periods.

At week 88, TmP/GFR data were available for 4 of 6 children in the burosumab continuation group and 14 of 15 children in the crossover group. Mean (SD) TmP/GFR in the burosumab continuation group was 2.0 (0.3) mg/dL at baseline, 3.6 (0.8) mg/dL at week 64 (mean [SD] percentage change from baseline, +77.4%; [17.3%]), and 3.3 (0.8) mg/dL at week 88 (+65.0% [33.3%]); Figure 5C. In the crossover group, mean (SD) TmP/GFR was 2.1 (0.3) mg/dL at baseline, 2.0 (0.5) mg/dL at week 64 (mean [SD] percentage change from baseline, −0.2% [18.0%]), and 3.4 (0.6) mg/dL at week 88 (+66.4% [27.1%]).

Mean (SD) serum 1,25(OH)_2_D in the burosumab continuation group was 38.0 (21.4) pg/mL at baseline, 61.7 (12.7) pg/mL at week 64 (mean percentage change from baseline, +70.9% [61.2%]), and 52.2 (9.7) pg/mL at week 88 (+62.4% [72.2%]; Figure 5D). In the crossover group, mean (SD) serum 1,25(OH)_2_D was 36.6 (11.0) pg/mL at baseline, 40.0 (12.1) pg/mL at week 64 (mean [SD] percentage change from baseline, +21.6%; [45.3%]), and 60.1 (16.1) pg/mL at week 88 (+89.6% [120.9%]). In the burosumab continuation group, mean (SD) serum 1,25(OH)_2_D was 33.5 (5.8) ng/mL at baseline, 36.6 (22.2) ng/mL at week 64 (mean [SD] percentage change from baseline, +7.0% [51.9%]), and 36.5 (15.6) ng/mL at week 88 (+10.6% [45.5%]); Figure 5E. In the crossover group, mean (SD) serum 1,25(OH)_2_D was 36.3 (8.9) ng/mL at baseline, 34.5 (10.3) ng/mL at week 64 (mean [SD] percentage change from baseline, −0.1% [40.2%]), and 30.7 (7.3) ng/mL at week 88 (−8.8% [34.4%]). No child in either treatment group had a serum 1,25(OH)_2_D level in the range associated with vitamin D deficiency rickets and osteomalacia (ie, <10 ng/mL) at any assessment. Most children had normal 1,25(OH)_2_D levels (>20 ng/mL or 50 nmol/L) throughout the study; however, one child in the burosumab continuation group and three in the crossover group had below normal values at individual time points. In the burosumab continuation group, one child had serum 1,25(OH)_2_D between 10 and < 20 ng/mL at week 16, and in the crossover group, three children had serum 1,25(OH)_2_D between 10 and < 20 ng/mL (week 40, *n* = 2; week 64, *n* = 1; week 76, *n* = 1; week 88, *n* = 1).

Throughout both phases of the study, mean intact parathyroid hormone (iPTH) was lower among children who received burosumab compared with conventional therapy except for the week 32 time point, when mean iPTH was slightly higher on burosumab than conventional therapy (Figure 5F). Plasma iPTH was within the normal range (14–72 pg/mL) in eight (38%) children (burosumab continuation, *n* = 3; crossover, *n* = 5) at all time points during the observation period, was above the ULN at least once in 11 (52%) children (burosumab continuation, *n* = 2; crossover, *n* = 9), and was below the ULN at least once in four (19%) children (burosumab continuation, *n* = 2; crossover, *n* = 2). The maximum observed iPTH value was 98 pg/mL (140% of the ULN) in the burosumab continuation group, and 175 pg/mL (240% of the ULN) in the crossover group, which occurred at week 64 before switching to burosumab.

**Figure 6:**
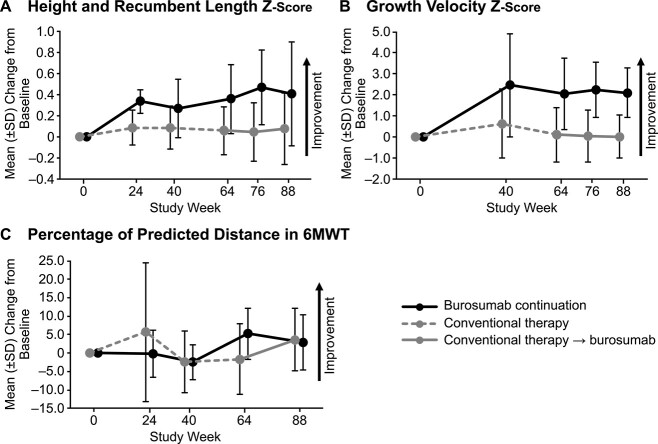
Mean (SD) change from baseline to 88 in recumbent length and standing height *Z*-score (A), growth velocity *Z*-score (B), and the percentage of predicted distance walked in the 6MWT (C). 6MWT, 6-minute walk test.

Among children in the burosumab continuation group, mean (SD) standing height and recumbent length *Z*-score improved from −2.79 (1.33) at baseline to −2.43 (1.29) at week 64 (mean [SD] change from baseline, +0.36 [0.33]) and to −2.38 (1.22) at week 88 (mean [SD] change from baseline, +0.41 [0.50]); Figure 6A. In the burosumab continuation group, 88% of the increase in mean standing height and recumbent length *Z*-score over 88 weeks of the study occurred between baseline and week 64, and 12% occurred between weeks 64 and 88. In the crossover group, mean (SD) standing height and recumbent length *Z*-score changed from −2.07 (1.01) at baseline to −2.01 (1.02) at week 64 (mean change from baseline, +0.06 [0.23]) and to −1.99 (1.14) at week 88 (mean [SD] change from baseline, +0.08 [0.34]); Figure 6A. There was a relatively linear increase in mean standing height and recumbent length in the crossover group, with 75% of the increase in occurring between baseline and week 64, and 25% occurring between weeks 64 and 88. Overall, the impact of burosumab on standing height and recumbent length *Z*-score was greater in the burosumab continuation group than in the crossover group.

Growth velocity *Z*-scores were calculated for the intervals of baseline to week 64 and baseline to week 88. In the burosumab continuation group, mean (SD) growth velocity *Z*-scores improved from −1.78 (1.24) at baseline to +0.27 (1.40) at week 64 (mean [SD] change from baseline, +2.05 [1.70]) and to +0.33 (1.19) at week 88 (mean [SD] change from baseline, +2.11 [1.18]); Figure 6B. In the burosumab continuation group, 97% of the increase in mean growth velocity *Z*-score over 88 weeks of the study occurred between baseline and week 64. In the crossover group, mean (SD) growth velocity *Z*-scores improved from −0.90 (1.14) at baseline to −0.78 (0.93) at week 64 (mean [SD] change from baseline, +0.12 [1.29]) and to −0.88 (0.82) at week 88 (mean [SD] change from baseline, +0.02 [1.02]); Figure 6B. In the crossover group, the mean growth velocity *Z*-score increased by 13% between baseline and week 64 but decreased by 11% from week 64 to week 88.

The 6MWT was conducted in children who were age 5 years and older at baseline (burosumab continuation, *n* = 3; crossover, *n* = 9). Children in the burosumab continuation group retained improvement from baseline in the percentage of predicted distance walked in the 6MWT, with a mean (SD) increase from 78.3% (13.3%) at baseline to 83.5% (16.6%) at week 64 (mean [SD] change from baseline, +5.3% [6.9%]) and to 81.2% (12.8%) at week 88 (+2.9% [7.5%]); Figure 6C. Children in the crossover group had increases in the percentage of predicted distance walked in the 6MWT, with a mean (SD) decrease from 86.1% (14.0%) at baseline to 84.5% (21.0%) at week 64 (mean [SD] change from baseline: −1.6% [9.5%]) and an increase to 89.7% (11.8%) at week 88 (mean [SD] change from baseline: +3.7% [8.5%]).

### Safety and tolerability

Safety and laboratory data are reported for 21 children with available radiographic assessments through study week 88 of the extension period. During the extension period, treatment-emergent AEs (TEAEs) were reported in 6 (100%) children in the burosumab continuation group and 14 (93%) children in the crossover group (Table 2). The AE profiles were similar during the randomized and extension periods, with no new safety signals identified during the extension period. Through the end of the study, the most frequent (occurring in ≥20%) TEAEs in both treatment groups (*n* = 21) included pyrexia (48%), nasopharyngitis (43%), injection site erythema (38%), arthralgia (29%), cough (29%), injection site reaction (29%), vomiting (29%), nausea (24%), and pain in extremity (24%). The frequency of injection site reaction events occurring with burosumab treatment was similar between the two groups during the extension period (burosumab continuation group, 50%; crossover group, 47%) and the randomized period (67%). The frequency of hypersensitivity on burosumab was lower during the extension period (burosumab continuation, 17%; crossover group, 0%) than during the randomized period (33%).

**Table 2:** Summary of AEs recorded during the randomized period and open-label extension period.

	**Randomized period (weeks 0–64)** ^**a**^		**Open-label extension period (weeks 64–88)** ^**a**^	
	**Conventional therapy (*n* = 15)**	**Burosumab (*n* = 6)**	**Crossover (*n* = 15)**	**Burosumab continuation (*n* = 6)**
Patients with any treatment-emergent AE, *n* (%)	14 (93)	6 (100)	14 (93)	6 (100)
Patients with any treatment-related AE, *n* (%)	3 (20)	5 (83)	8 (53)	3 (50)
Patients with any serious treatment-emergent AE, *n* (%)	0	0	0	0
Patients with any serious treatment-related AE, *n* (%)	0	0	0	0
Patients with grade 3 or 4 AEs, *n* (%)	0	1 (17)	0	0
Predefined AEs of interest, *n* (%)				
Injection site reactions	0	4 (67)	7 (47)	3 (50)
Hypersensitivity	5 (33)	2 (33)	0	1 (17)
Hyperphosphatemia	0	0	0	0
Ectopic mineralization	0	0	0	0
Restless legs syndrome	0	0	0	0
Treatment-emergent AEs occurring in ≥10% of patients,^b^*n* (%)				
Pyrexia	5 (33)	5 (83)	4 (27)	3 (50)
Nasopharyngitis	10 (67)	3 (50)	4 (27)	2 (33)
Injection site erythema	0	3 (50)	5 (33)	2 (33)
Arthralgia	4 (27)	4 (67)	2 (13)	1 (17)
Cough	5 (33)	2 (33)	4 (27)	1 (17)
Injection site reaction	0	3 (50)	2 (13)	3 (50)
Vomiting	5 (33)	2 (33)	4 (27)	1 (17)
Nausea	1 (7)	2 (33)	2 (13)	1 (17)
Pain in extremity	5 (33)	2 (33)	3 (20)	1 (17)
Headache	3 (20)	2 (33)	2 (13)	0
Nasal congestion	1 (7)	1 (17)	1 (7)	2 (33)
Rhinorrhea	2 (13)	0	3 (20)	1 (17)
Constipation	0	1 (17)	2 (13)	0
Dental caries	1 (7)	2 (33)	0	1 (17)
Injection site pruritus	0	1 (17)	2 (13)	0
Tooth abscess	1 (7)	1 (17)	1 (7)	1 (17)
Upper abdominal pain	2 (13)	1 (17)	2 (13)	0
Abdominal discomfort	1 (7)	1 (17)	1 (7)	0
Ear discomfort	0	1 (17)	0	1 (17)
Ear infection	3 (20)	0	1 (7)	1 (17)
Injection site bruising	0	0	2 (13)	0
Injection site urticaria	0	2 (33)	0	0
Oropharyngeal pain	0	0	1 (7)	1 (17)
Seasonal allergy	1 (7)	1 (17)	1 (7)	0
Sinus congestion	0	0	1 (7)	1 (17)
Streptococcal pharyngitis	1 (7)	0	1 (7)	1 (17)
Toothache	1 (7)	0	1 (7)	1 (17)
Tooth loss	0	0	1 (7)	1 (17)

AE, adverse event.

^a^Data are shown for children with available radiographic assessments through week 88.

^b^Treatment-emergent AEs occurring in ≥10% of both treatment groups (*n* = 21) from baseline through end of study.

Consistent with the randomized period, all 21 children (100%) during the extension period had mild or moderate (grades 1 or 2) TEAEs (Table 2). No grade ≥ 3 TEAEs or serious TEAEs were reported during the extension period. One child in the burosumab continuation group had experienced grade 3 viral gastroenteritis during the randomized period that was not related to burosumab treatment. None of the 21 children had serious AEs during the randomized or extension periods.

During the extension period, treatment-related AEs were reported in three (50%) children in the burosumab continuation group and eight (53%) children in the crossover group. During the randomized period, treatment-related AEs occurred in five (83%) children who received burosumab and three (20%) children who received conventional therapy. Across the study, the most frequent (occurring in ≥10% of children) AEs related to treatment with burosumab were injection site erythema (38%), injection site reaction (29%), injection site pruritus (14%), injection site bruising (10%), injection site swelling (10%), and injection site urticaria (10%). No children discontinued treatment due to AEs, and there were no fatal AEs. The frequency of dental abscesses was the same in the randomized and extension periods. Overall, the incidence was slightly higher in the burosumab group compared to conventional therapy, and slightly higher in the burosumab continuation group compared to those who switched from conventional therapy to burosumab during the extension period.

During the randomized period, there were no children in either the burosumab continuation or crossover groups with increases in nephrocalcinosis scores. During the extension period, one child (7%) in the crossover group had a single-point nephrocalcinosis score increase; no other increases occurred (Supplementary Figure 1).

## Discussion

In this open-label extension of the 64-week phase 3 randomized controlled trial of burosumab vs conventional therapy in children with XLH,^10^ we describe the response to burosumab in those children with XLH who continued burosumab through 88 weeks and in those who crossed over to burosumab at week 64 after initially receiving conventional therapy. Given that all children in this study had 88-week comparisons with baseline, the data provide novel information concerning clinical and safety outcomes.

Consistent with the results of the randomized period of the trial,^10^ the findings in the crossover group demonstrated that the biochemical response to FGF23 blockade was both immediate and robust compared with prior conventional treatment. In the crossover group, serum phosphorus concentrations normalized rapidly, affirming that improved phosphate reabsorption with burosumab is rapid and sustained, even when prior conventional therapy has stopped. Similarly, the increase in serum 1,25(OH)_2_D was also immediate with crossover to burosumab. The highest individual burosumab dose was 1.27 mg/kg in the randomized period and 1.27 mg/kg through week 88 of the extension period. There were no occurrences of hyperphosphatemia observed in either the burosumab continuation or crossover groups during the randomized or extension periods. The importance of this observation cannot be underestimated, as it continues to affirm that despite the ongoing burosumab administration at doses higher than those at initiation, and that despite additional evidence that burosumab maintains the increases in serum phosphate levels observed in children over the longer term, there does not appear to be a narrow therapeutic threshold causing a tendency in some patients to exceed the ULN for age. This is highly relevant information for clinicians who may endeavor to safely optimize clinical outcomes by increasing the burosumab dose to the highest levels observed in this study.

Although rickets healing and improvement in lower limb deformity occurred predominantly during the first 64 weeks of treatment with burosumab,^10^ both continued with burosumab treatment during the open-label extension. In the crossover group, rickets healing was markedly improved after only 22 weeks of treatment with burosumab, achieving a mean (SD) RGI-C rickets total score of +1.89 (0.35) at week 88 (compared with +2.00 in the continuation group after 64 weeks of treatment). Similarly, 100% of the improvement in total RSS in the burosumab continuation group occurred by week 64, whereas 61% of the improvement in the crossover group occurred after initiating burosumab at week 64. By the end of the 88-week extension period, all children in the burosumab continuation group and 87% of the children in the crossover group had substantial healing of rickets (defined as RGI-C rickets total score ≥ +2). Improvements in lower limb deformity on burosumab were greatest during the first year, but further improvements continued up to 88 weeks. Furthermore, the wrist and knee growth plate findings via the RGI-C score suggest that metaphyseal concavity may resolve more slowly than metaphyseal fraying, lucency, and separation. Long-term safety and rickets healing have also been documented in children with XLH aged 5–12 years who received burosumab Q2W or Q4W for 160 weeks in the extension of an open-label phase 2 study.^20^

Elevated serum ALP, a biochemical hallmark of rickets and osteomalacia, is typical in children with XLH.^21^ Clinically significant declines in serum ALP were associated with the improvements in rickets and lower limb deformity observed early during treatment with burosumab and with those that continued beyond week 64. By week 88 in the burosumab continuation group, serum ALP was within the normal range for age and gender in 50% of children. Overall, our observations affirm that improvements in three clinical indicators of disease severity—rickets severity, lower limb deformity, and ALP—show a significant early response to burosumab as previously shown in the phase 3 trial.^10^ Furthermore, although these positive changes are substantial in the first year, they further improve for up to 88 weeks of treatment. Further investigation is required to determine whether these clinical indicators will improve beyond 88 weeks or plateau, and whether these outcomes are influenced by factors such as age at diagnosis or treatment initiation, severity of disease manifestations at baseline, or specific *PHEX* mutations.

Although improvements in linear growth during the randomized period of the trial persisted with burosumab continuation during the extension period, further height/recumbent length *Z*-score increases were not observed. This was surprising, given that ongoing improvement in rickets severity and lower limb deformity with burosumab were observed to week 88, along with rapid and sustained improvement in phosphate homeostasis and a progressive decline in serum ALP. However, the observation that children in the burosumab continuation group maintained increases in height *Z*-score and growth velocity *Z*-score compared with baseline indicates continued growth at an appropriate rate for age, rather than plateauing or a declining in growth rate. The limited *Z*-score increases may be attributed, at least in part, to the observed decline in growth velocity observed in normal children after 2 years of age that lasts until puberty,^17^^,^^22^ as well as greater declines in growth velocity after 1 year of age in children with XLH compared with healthy children.^23^ Notably, when we evaluated children with XLH younger than 5 years vs those aged 5–12 years, we found that height/recumbent length *Z*-scores appeared to decline in those under 5 years receiving conventional therapy during the first 64 weeks, but increased in that age group when receiving burosumab.^24^

Importantly, children in the crossover group had a less robust growth response to 22 weeks of treatment with burosumab than did children in the burosumab continuation group during the first 24 weeks of treatment in the randomized period. This difference could reflect, at least in part, the relatively small sample size of children in this sub-analysis (*n* = 21) and the lack of strict randomization or sufficient statistical power to test differences in observed outcomes. Several biological factors may have contributed to the attenuated growth response in the crossover group. First, immediately before burosumab, children in the crossover group appeared to have a less severe phenotype than in the burosumab continuation group, as indicated by the lower average total RSS and the higher average height *Z*-scores. Hence, it may be more difficult to document a change in height *Z*-scores because outcomes in question already approximate the mean. Second, the crossover group averaged 66 weeks older than the burosumab continuation group at burosumab initiation, perhaps affecting growth response, as that age difference also affects growth rates in healthy children.^17^^,^^22^

Improvements in mobility were maintained in the burosumab continuation group, with a modest 2.9% increase in the mean percentage of predicted 6MWT distance from baseline to week 88. In the crossover group, the mean percentage of predicted distance walked decreased by only 1.6% with 64 weeks of conventional therapy, followed by a reversal of the downward trajectory following burosumab initiation, with a mean increase of 3.7% from baseline to week 88 (including a 5.3% increase from weeks 64 to 88). The improved mobility in the crossover group is noteworthy considering the relatively short duration of burosumab (22 weeks) when compared with the 9% increase in the percentage after 64 weeks of burosumab in the full study population during the randomized period (*N* = 61).^10^

During this extension period, treatment with burosumab was well tolerated, and there were no new safety findings compared with the randomized period and with prior studies.^10^^,^^12^^,^^25^ The most common AEs were typical of normal pediatric illnesses (eg, pyrexia, cough, and vomiting) and complaints frequently reported in pediatric XLH (eg, leg pains), and of those associated with a subcutaneous antibody therapy (eg, injection site reactions). Importantly, no child discontinued the study due to AEs, and the incidence and severity of nephrocalcinosis and hyperparathyroidism did not emerge as safety concerns. No cases of worsening nephrocalcinosis were observed in the burosumab group during the randomized period, whereas one child in the crossover group had a one-unit increase in nephrocalcinosis score during the extension period. We also observed that mean plasma iPTH was lower in children who received burosumab than in those who received conventional therapy at all time points during the trial except for week 32, when mean plasma iPTH in the burosumab-treated group was slightly higher than on conventional therapy but nevertheless within the normal range. This increase in mean plasma iPTH at week 32 corresponded with a concomitant rise in average serum phosphorus (the latter, for reasons that remain unclear).

Several limitations of this study merit consideration. First, this was a subgroup analysis of the original phase 3, randomized controlled trial and was therefore not powered to formally assess within- or between-group differences in outcomes. Importantly, however, the relationship between improved outcomes and the timing of burosumab initiation could be evaluated, since the children in the crossover group received conventional therapy in a well-controlled clinical trial setting that may have led to a more compliant control group before these participants crossed over to burosumab treatment. The growth velocity *Z*-scores at baseline had to be calculated based on pre-study height values, thereby adding potential variability to the baseline growth velocity estimates, whereas the growth velocity calculated during the trial was based on standardized height measurements. Several questions were unanswered in this study, including the impact of burosumab on adult height and body proportion, need for orthopedic surgery, and complications of XLH in adulthood (eg, enthesopathy, osteoarthritis, and spinal stenosis). Finally, the effects of burosumab initiated in the first few years of life on prevention of bone deformity, craniosynostosis, and modulation of dental phenotype remain to be investigated.

In summary, improvements in rickets healing, lower limb deformity, and ALP were evident, whether children continued burosumab or initiated burosumab therapy after receiving conventional therapy for 64 weeks in a clinical trial setting. Rapid improvement in phosphate metabolism and positive effects on the percentage of the predicted distance walked were observed in children who crossed over from conventional therapy to burosumab and were maintained among children who continued burosumab. The positive effect of burosumab on growth in the first 64 weeks was maintained to week 88 in the burosumab continuation group; however, the crossover group did not exhibit an increase in standing height/recumbent length *Z*-score after the initiation of burosumab, likely due to the limited duration of burosumab therapy (22 weeks) in this crossover group. Further investigation in a larger cohort is necessary to better understand the variability in the growth response to burosumab across different ages and disease severities, and the impact of age at burosumab initiation on growth. Some of this variability in response likely results from the normal decrease in growth velocity in children from the age of two years until the prepubertal period. Overall, burosumab was well tolerated and there were no new safety signals identified in this open-label extension period.

## Supplementary Material

Ward_et_al-Supplemental_Tables_ziad001

## Data Availability

All relevant data are available from the authors upon reasonable request.
