# Supplementary material for: Burosumab vs conventional therapy in children with X-linked hypophosphatemia: results of the open-label, phase 3 extension period
Source: JBMR Plus. 2024 Jan 4;8(1):ziad001. doi: 10.1093/jbmrpl/ziad001 (PMC11059996; doi:10.1093/jbmrpl/ziad001)
Supplement: Ward_et_al-Supplemental_Tables_ziad001 [file ward_et_al-supplemental_tables_ziad001.docx]

**Supplemental Table 1. Summary of RGI-C Scores by Time Point**

|  | **Week 40 Randomized Period** | |  | **Week 64 Randomized Period** | |  | **Week 88 Open-Label Extension Period** | |
| --- | --- | --- | --- | --- | --- | --- | --- | --- |
|  | **Conventional Therapy (N=15)** | **Burosumab (N=6)** |  | **Conventional Therapy (N=15)** | **Burosumab  (N=6)** |  | **Crossover (N=15)** | **Burosumab Continuation (N=6)** |
| Mean (SD) total score | 0.73 (0.70) | 1.83 (0.41) |  | 0.78 (0.75) | 2.00 (0) |  | 1.89 (0.35) | 2.11 (0.27) |
| Mean (SD) knee score | 0.69 (0.61) | 1.72 (0.39) |  | 0.80 (0.78) | 2.00 (0) |  | 1.87 (0.35) | 2.06 (0.14) |
| Mean (SD) wrist score | 0.71 (0.83) | 2.00 (0.56) |  | 0.80 (0.82) | 2.28 (0.53) |  | 2.02 (0.37) | 2.17 (0.46) |
| Mean (SD) lower limb deformity score | 0.20 (0.49) | 0.72 (0.68) |  | 0.20 (0.73) | 1.44 (0.75) |  | 0.73 (0.82) | 1.61 (0.91) |

RGI-C, Radiographic Global Impression of Change; SD, standard deviation.

**Supplemental Table 2. Number of Children with Improvements from Baseline in Specific Wrist and Knee Rickets Radiographic Abnormalities per RGI-C**

|  | **Week 40 Randomized Period** | |  | **Week 64 Randomized Period** | |  | **Week 88 Open-Label Extension Period** | |
| --- | --- | --- | --- | --- | --- | --- | --- | --- |
| **Number With Improvement at Time Point / Number with Abnormality at Baseline (%)** | **Conventional Therapy (N=15)** | **Burosumab (N=6)** |  | **Conventional Therapy (N=15)** | **Burosumab  (N=6)** |  | **Crossover (N=15)** | **Burosumab Continuation (N=6)** |
| Radius | | | | | | | | |
| Metaphyseal lucency | 9/14 (64) | 6/6 (100) |  | 7/15 (47) | 6/6 (100) |  | 14/15 (93) | 6/6 (100) |
| Metaphyseal/epiphyseal separation | 5/11 (46) | 5/6 (83) |  | 7/15 (47) | 6/6 (100) |  | 14/14 (100) | 6/6 (100) |
| Metaphyseal fraying | 4/9 (44) | 5/5 (100) |  | 4/7 (57) | 6/6 (100) |  | 11/11 (100) | 6/6 (100) |
| Metaphyseal concavity | 5/8 (63) | 6/6 (100) |  | 4/10 (40) | 6/6 (100) |  | 8/8 (100) | 6/6 (100) |
| Ulna | | | | | | | | |
| Metaphyseal lucency | 8/15 (53) | 5/6 (83) |  | 8/15 (53) | 5/6 (83) |  | 15/15 (100) | 6/6 (100) |
| Metaphyseal/epiphyseal separation | 4/7 (57) | 3/3 (100) |  | 4/8 (50) | 3/3 (100) |  | 8/9 (89) | 3/3 (100) |
| Metaphyseal fraying | 4/12 (33) | 4/4 (100) |  | 7/12 (58) | 5/5 (100) |  | 13/13 (100) | 5/5 (100) |
| Metaphyseal concavity | 7/15 (47) | 6/6 (100) |  | 8/15 (53) | 6/6 (100) |  | 14/14 (100) | 6/6 (100) |
| Femur |  |  |  |  |  |  |  |  |
| Metaphyseal lucency | 9/15 (60) | 6/6 (100) |  | 12/15 (80) | 6/6 (100) |  | 15/15 (100) | 6/6 (100) |
| Metaphyseal/epiphyseal separation | 4/15 (27) | 6/6 (100) |  | 9/15 (60) | 6/6 (100) |  | 15/15 (100) | 6/6 (100) |
| Metaphyseal fraying | 7/15 (47) | 6/6 (100) |  | 11/15 (73) | 6/6 (100) |  | 14/15 (93) | 6/6 (100) |
| Metaphyseal concavity | 5/15 (33) | 6/6 (100) |  | 10/15 (67) | 6/6 (100) |  | 15/15 (100) | 6/6 (100) |
| Tibia | | | | | | | | |
| Metaphyseal lucency | 11/15 (73) | 5/6 (83) |  | 12/15 (80) | 6/6 (100) |  | 15/15 (100) | 6/6 (100) |
| Metaphyseal/epiphyseal separation | 7/15 (47) | 6/6 (100) |  | 6/15 (40) | 6/6 (100) |  | 15/15 (100) | 6/6 (100) |
| Metaphyseal fraying | 8/15 (53) | 5/6 (83) |  | 7/14 (50) | 6/6 (100) |  | 14/15 (93) | 5/5 (100) |
| Metaphyseal concavity | 8/15 (53) | 6/6 (100) |  | 11/15 (73) | 6/6 (100) |  | 14/15 (93) | 6/6 (100) |
| Fibula |  |  |  |  |  |  |  |  |
| Metaphyseal lucency | 3/5 (60) | 4/5 (80) |  | 3/8 (38) | 3/4 (75) |  | 9/11 (82) | 6/6 (100) |
| Metaphyseal/epiphyseal separation | 2/3 (67) | 3/3 (100) |  | 3/5 (60) | 1/1 (100) |  | 4/5 (80) | 3/3 (100) |
| Metaphyseal fraying | 0/2 (0) | 2/2 (100) |  | 1/1 (100) | 2/2 (100) |  | 4/4 (100) | 1/1 (100) |
| Metaphyseal concavity | 0/3 (0) | 2/2 (100) |  | 2/6 (33) | 2/2 (100) |  | 6/7 (86) | 2/3 (67) |

m, number of subjects with abnormality at baseline; n, number of subjects with improvement in the abnormality; RGI-C, Radiographic Global Impression of Change.
